# Analysis of percutaneous kyphoplasty under different types of anesthesia for the treatment of multiple osteoporotic vertebral fractures

**DOI:** 10.1186/s12891-020-03778-1

**Published:** 2020-11-12

**Authors:** Shuai Zhang, Shuang Xu, Jin Yang, Song Wang, Qing Wang

**Affiliations:** Department of Orthopedics, The Affiliated Hospital of Southwest Medical University, NO. 25 Taiping Street, Luzhou City, 646000 Sichuan China

**Keywords:** Percutaneous kyphoplasty, Multiple osteoporotic vertebral fractures, General anesthesia, Local anesthesia

## Abstract

**Background:**

Surgeons recognize that using percutaneous kyphoplasty (PKP) under local anesthesia to treat osteoporotic vertebral fracture (OVF) prevents interference with the general situation of elderly patients suffering from multiple organ dysfunction. Surgeons can directly assess whether nerve injury occurs while the patient is awake. However, when patients with multiple osteoporotic vertebral fractures (m-OVFs) receive local anesthesia, fluoroscopy time often has to be increased, the operative time has to be extended, or the operation has to be terminated because of discomfort related to body posture. No relevant study has thus far been conducted on the type of anesthesia to administer to patients undergoing PKP for m-OVFs. This study aimed to determine which of the two types of anesthesia is more suitable for PKP for m-OVFs.

**Methods:**

A retrospective study was conducted involving 159 patients who underwent PKP for m-OVFs from January 2016 to January 2020; 81 patients underwent PKP under general anesthesia (Group G), and 78 patients underwent PKP under local anesthesia (Group L). Clinical and adverse events were compared between the two groups.

**Results:**

The intraoperative mean arterial pressure, average heart rate, average fluoroscopy times of each vertebral body, and operative time were less in Group G than in Group L. The visual analog scale (VAS) score was significantly lower after than before the operation. The anterior vertebral height (AVH), middle vertebral height (MVH), and kyphotic angle (KA) were significantly improved in both groups postoperatively. The improvement in VAS score, AVH, MVH, and KA in Group G were higher than those in Group L. No significant difference in the incidence of complications was observed between the two groups.

**Conclusion:**

PKP under either general anesthesia or local anesthesia was reliable. Compared with PKP under local anesthesia, PKP under general anesthesia could more reliably maintain the stability of vital signs, alleviate preoperative pain in patients, and attain a better orthopedic effect. Moreover, the latter does not increase the complications of patients with m-OVFs. However, the high medical expense of PKP under general anesthesia is a factor to consider when choosing the type of anesthesia.

## Background

The World Health Organization considers osteoporosis to be second only to cardiovascular diseases as a critical health problem [1]. Minor injuries in daily activities can cause fragility fractures, which are attributable to decreased bone mass and strength, as well as increased bone fragility. The most common fracture is osteoporotic vertebral fracture (OVF) [2–4]. OVF often causes severe pain, spinal deformity, skeletal muscle function deficit, and dysmobility syndrome, among others. These problems arise particularly when multiple vertebral body fractures (m-OVFs) occur simultaneously, often rendering ambulatory patients at increased risk of poor physical performance, higher frequency of falls, and further fracture risk [5, 6]. To increase the ability of the patient to perform daily activities and relieve pain, braces can typically be used to protect, strengthen core muscle strength exercises, and orally take nonsteroidal anti-inflammatory analgesics or opioid analgesics [7, 8]. To delay the progress of osteoporosis and improve bone metabolism, several studies have reported that subcutaneous injection of denosumab or teriparatide and standardized oral bisphosphonates can achieve satisfactory clinical effects [9, 10]. Percutaneous kyphoplasty (PKP) can effectively treat patients of OVF who cannot tolerate pain or do not improve from conservative treatment [11–13].

Surgeons recognize the advantage of PKP under local anesthesia for the treatment of OVF in preventing interference with the general situation of elderly patients with multiple organ dysfunction. Surgeons can directly assess whether the nerve injury occurs while the patient is awake. Moreover, patients who receive local anesthesia do not need postoperative resuscitation and can get out of bed early, which is conducive to postoperative rehabilitation [14–16]. However, when patients with m-OVFs receive local anesthesia, fluoroscopy time often has to be increased, the operative time has to be extended, or the operation has to be terminated because of body posture-related discomfort, excessive local anesthetic drugs, the toxic reaction of bone cement, and abrupt fluctuation of vital signs. The literature shows that the type of anesthesia does not affect the efficacy of PKP for a single-level OVF, and general anesthesia increases the incidence of complications and medical costs [14, 17, 18]. To the best of our knowledge, no studies have been reported on the choice of the type of anesthesia to administer to patients undergoing PKP for m-OVFs. This study aimed to determine whether PKP for m-OVFs is more suitable under general or local anesthesia.

## Methods

The study was authorized by the Ethics Committee of the Affiliated Hospital of Southwest Medical University.

### Patient population

All patients underwent PKP at the Department of Orthopedics of the Affiliated Hospital of Southwest Medical University from January 2016 to January 2020. All data were retrospectively reviewed based on medical records and billing statements. The inclusion criteria were as follows: (1) clinical manifestations of different degrees of waist and back pain, pain aggravated after turning over and getting up, no relief from conservative treatment over 2 weeks, and positive percussion pain of the corresponding spinous process; (2) no signs or symptoms of spinal cord or nerve root damage in the corresponding fracture segment before the operation; (3) presence of osteoporosis as determined by dual energy X-ray absorptiometry; (4) m-OVFs on magnetic resonance imaging (MRI) with low signal intensity on T1-weighted imaging, high signal intensity on T2-weighted imaging, and high signal intensity on short TI inversion recovery; and (5) simultaneous PKP procedures on 3 or 4 vertebral bodies. The exclusion criteria were as follows: (1) coagulopathy in the patient or administration of anticoagulant therapy to the patient; (2) patients assigned to class IV or V under the American Society of Anesthesiologists classification system; (3) serious spinal instability caused by pedicle fracture; (4) symptomatic neurologic injury; (5) non-OVF conditions, such as tumors or infectious diseases, as confirmed by pathological examination; (6) Localized infection at the operative site and/or systemic sepsis; (7) unilateral puncture unable to achieve bone cement isocentric distribution and needs to supplement the contralateral puncture.

In accordance with the inclusion and exclusion criteria, 159 patients were recruited in this study, with 81 patients assigned to Group G (the general anesthesia group) and 78 patients assigned to Group L (the local anesthesia group). The baseline data of the patients were collected, including but not limited to age, gender, occupation, and bone mineral density. The volume of bone cement injected into each vertebral body and fracture was also collected.

### Anesthesia and surgical procedures

Group G received general anesthesia via endotracheal intubation. General anesthesia was induced using etomidate 2 mg/kg, sufentanil 0.2 μg/kg, and cisatracurium 0.15 mg/kg during the operation. Moreover, 2–3% sevoflurane was inhaled continuously, and propofol 2 mg/(kg·h) was continuously pumped into the vein. The patients were then sent to the postanesthesia care unit for observation after extubation. For group L, 1% lidocaine was used for local infiltration anesthesia. If the intraoperative systolic blood pressure was higher than 180 mmHg, nicardipine 0.03–0.3 mg/(kg·h) was continuously pumped to control blood pressure.

With fluoroscopy visualization, transpedicular (in the lumbar vertebrae) or extrapedicular (in the thoracic vertebrae) puncture was performed unilaterally. After reaching the posterior margin of the vertebral body, the bone needle was replaced with a working cannula. A balloon with a radiopaque medium was inserted into the fractured vertebral body to restore the damaged vertebral body until adequate height restoration and kyphosis correction were obtained. The balloon was then deflated and withdrawn, and the resultant intravertebral cavity was filled with polymethylmethacrylate cement.

### Outcome measures

All patients underwent thoracolumbar anteroposterior and lateral plain film radiography, whole-spine MRI, and bone mineral density (dual energy X-ray absorptiometry) preoperatively. All patients underwent PKP and pathological biopsy simultaneously. The thoracolumbar plain films were routinely reviewed 1 d after the operation. The effects of the different types of anesthesia on the intraoperative conditions of the patients were evaluated by comparing the mean arterial pressure, average heart rate, blood loss, average fluoroscopy times of each vertebral body, and operative time (from the first vertebral puncture to the end of wound dressing) between the groups.

VAS scores (range: 0–10, with 0 indicating no pain and 10 indicating unbearable, severe pain) were recorded before and after the operation. Following the method proposed by Lee and Kuklo [19, 20], the anterior vertebral height (AVH), middle vertebral height (MVH), and kyphotic angle (KA) were measured before and after the operation. To evaluate the effects of the different types of anesthesia on clinical efficacy, the following were compared between the two groups: improvement in visual analog scale (VAS) scores [(preoperative VAS score - postoperative VAS score)/preoperative VAS score]*100%, recovery rate of the AVH [(postoperative AVH – preoperative AVH)/preoperative AVH]*100%, recovery rate of the MVH[(postoperative MVH – preoperative MVH)/preoperative MVH]*100%, and the improvement rate of KA[(preoperative KA – postoperative KA)/preoperative KA] * 100%. To evaluate the effects of the different types of anesthesia on postoperative recovery, the incidence of complications was also compared between the two groups. Total expenditure and the expenditure for anesthesia, device, drug, and nursing were determined. Similarly, the expenses after the operation were calculated. All costs were expressed in dollars.

### Statistical analysis

All data were statistically analyzed using SPSS 19.0 (Chicago, IL, USA). The classified variable was calculated using a chi-squared test and Fisher’s exact test. Continuous variables, presented as mean standard deviation, were calculated using the Mann–Whitney test and paired or unpaired t-test with or without Welch’s correction. All statistical data are presented in tabular form. *P* < 0.05 indicates that the difference is statistically significant.

## Results

No difference in baseline information was found between the two groups (*P* > 0.05). The volume of bone cement injected into each vertebral body was larger in Group G than in Group L (*P* < 0.05). The intraoperative mean arterial pressure, average heart rate, average fluoroscopy times of each vertebral body, and operative time were significantly higher in Group L than Group G (*P* < 0.05); however, no significant difference in intraoperative blood loss was found between the two groups (*P* > 0.05) (Table 1).

**Table 1:** Comparison of patients’ characteristics

Characteristics	Group G (*n* = 81)	Group L (*n* = 78)	Test value	*P* value
**Age**	72.67 ± 8.46	73.10 ± 7.22	−0.349	0.728
**Female**	52 (64.2)	51 (65.4)	0.025	0.876
**Occupation**				
Worker	18	16		
Farmer	16	17		
Unemployed	8	7		
Technician	9	11		
Retiree	22	21		
Others	8	6	0.667	0.985
**BMD**	−3.61 ± 0.80	−3.52 ± 0.66	0.768	0.444
**Fracture level**				
T_7_ ~ T_10_	94	102		
T_11_ ~ L_2_	118	114		
L_3_ ~ L_5_	72	65	4.557	0.919
**Volume of bone Cement injected into each Vertebra (ml)**	6.32 ± 0.95	5.46 ± 1.02	10.328	0.000
**Mean arterial Pressure (mmHg)**	101.1 ± 7.49	134.2 ± 12.95	−19.809	0.000
**The average of heart beats**	64.9 ± 4.03	88.1 ± 8.77	−21.495	0.000
**Amount of Bleeding (ml)**	67.2 ± 22.37	69.6 ± 21.41	−0.067	0.504
**Fluoroscopy times of each vertebra**	18.7 ± 1.70	24.4 ± 1.81	−20.368	0.000
**Time of operation (min)**	79.6 ± 14.37	94.5 ± 13.82	−6.663	0.000
**Complications**				
Bone cement	26 (9.2)	18 (6.4)	1.487	0.223
Neurological impairment	4 (4.9)	2 (2.6)	0.613	0.434
Vertebral infection	1 (0.4)	1 (0.4)	0.000	0.994
Cognitive dysfunction	7 (8.6)	2 (2.6)	2.732	0.098
Cardiopulmonary complications	4 (4.9)	1 (1.3)	1.733	0.188

The pain and VAS scores in the two groups were significantly reduced postoperatively (*P* < 0.05). However, the improvement in the VAS score was significantly more favorable in Group G than in Group L (*P* < 0.05). The heights of the vertebral body in the two groups were significantly recovered postoperatively (*P* < 0.05), but the recovery rate of the vertebral height in Group G was significantly preferable to that in Group L (*P* < 0.05). The KA in the two groups was significantly recovered postoperatively (*P* < 0.05), but the improvement rate of KA in Group G was significantly higher than that in Group L (*P* < 0.05) (Tables 2, 3).

**Table 2:** Comparison of VAS pain scores, AVH, MVH and KA of the vertebral body before, postoperation in group G and group L

	Pre-operation	Post-operation	Test value	*P* value
VAS pain scores				
Group G	7.21 ± 1.08	1.56 ± 0.67	40.008	0.000
Group L	7.15 ± 1.09	2.41 ± 0.84	30.318	0.000
AVH (mm)				
Group G	18.31 ± 2.75	22.69 ± 2.98	−18.175	0.000
Group L	17.83 ± 3.22	20.88 ± 3.38	−10.948	0.000
MVH (mm)				
Group G	17.85 ± 2.17	22.91 ± 2.78	−24.189	0.000
Group L	17.69 ± 2.50	20.83 ± 5.58	−10.371	0.000
KA (°)				
Group G	19.78 ± 2.67	14.29 ± 2.89	23.523	0.000
Group L	19.25 ± 3.27	15.56 ± 3.42	9.249	0.000

**Table 3:** Comparison of the improvement rate of VAS pain scores, AVH, MVH, and KA of the vertebral body before, postoperation in group G and group L

	The improvement rate of VAS pain scores	The improvement rate of AVH	The improvement rate of MVH	The improvement rate of KA
Group G	77.8 ± 10.57	24.5 ± 6.60	28.5 ± 6.34	28.2 ± 9.11
Group L	65.2 ± 14.49	17.6 ± 4.52	18.1 ± 4.27	20.2 ± 7.54
Test value	6.282	14.356	17.715	9.013
*P* value	0.000	0.000	0.000	0.000

Bone cement leakage occurred in 26 cases and 18 cases, nerve injury in 4 cases and 2 cases, vertebral infection in 1 case, cognitive dysfunction in 7 cases and 2 cases, cardiopulmonary complications in 4 cases and 1 case, respectively. No significant difference in the incidence of complications was determined between the two groups (*P* > 0.05) (Table 1). Leakage of the T10 vertebral bone cement into the spinal canal, compressing the spinal cord, was observed in 1 of 6 patients with a spinal cord injury in Group G. The patient suffered from numbness, pain, and fatigue on both lower limbs after the operation. The nerve function was not recovered after emergency bone cement removal. Grade C under the American Spinal Cord Injury Association was retained at discharge. The remaining 5 patients completely recovered after conservative treatment. Two patients suffered from postoperative vertebral infection, which was treated with bone cement removal, debridement, bone graft fusion, and internal fixation. However, 1 of 2 patients died of a secondary infection after the operation. The patients who suffered from postoperative cognitive dysfunction and cardiopulmonary complications recovered and were discharged after conservative treatment. The average medical expenditure incurred in Group G was 7247.8 ± 40.54 US$, and that in Group L was 6752.6 ± 37.21 US$. Hospitalization cost was significantly higher in Group G than Group L (*P* < 0.05).

## Discussion

To minimize the effect on the general situation of elderly patients, PKP under local anesthesia is an effective method for treating OVFs [14–18]. Liu et al. reported that local anesthesia for single vertebra PKP surgery can effectively relieve pain. Local anesthesia can provide the same clinical efficacy and spinal deformity correction as that of general anesthesia. Moreover, PKP surgery under local anesthesia can effectively reduce anesthesia-related complications such as cardiopulmonary system, allowing patients to get out of bed early and reduce hospitalization time and medical expenses [17]. Fang et al. indicated that the type of anesthesia administered does not affect the clinical efficacy of PKP [18]. With the increasing aging of the population, patients with m-OVFs are often encountered in the clinical setting. Previous studies have only included the single-level OVF patients in the study. The time required for a single OVF to undergo PKP surgery is short, and the requirements for the basic health of the patient are lower. The anesthesia method often only slightly affects the clinical efficacy of this type of patient. To the best of our knowledge, no research has thus far been reported on the choice of the type of anesthesia to administer to patients undergoing PKP for m-OVFs. In addition, the previous literature did not compare the intraoperative conditions under different anesthesia methods. The sharp fluctuations in the mean arterial pressure, heart rate, and other vital signs during the operation are often closely related to the occurrence of postoperative cognitive dysfunction and other complications. Moreover, the current study used the VAS score improvement rate, vertebral height improvement rate, and local kyphotic angle improvement rate to evaluate clinical efficacy for the first time, improving the accuracy of research results.

During surgery under general anesthesia, vital signs such as mean arterial pressure and heart rate can be maintained within a relatively stable range because of the continuous application of narcotic analgesics, narcotic sedatives, and muscle relaxants, combined with efficient respiratory ventilation management. Patients lose the perception of pain stimulation, and no significant fluctuation of blood pressure and heart rate due to insufficient local anesthesia occurs during the operation. Abrupt fluctuations of blood pressure and heart rate need to be avoided to reduce cardiovascular complications and acute cerebral infarction [21–23]. Relative to those under local anesthesia, the operative time and fluoroscopy exposure times are reduced under general anesthesia for multiple vertebral PKP mainly because the patient shows no frequent change in position due to body position-related discomfort nor stimulation of intraoperative pain. With a general muscle relaxant, the surgeon can more effectively and accurately operate on the patient. For multiple vertebral PKP under general or local anesthesia, postoperative pain can be significantly alleviated, and a satisfactory clinical effect can be achieved. However, this study found that the improvement rate of postoperative pain in the local anesthesia group was lower than that in the general anesthesia group, and this difference could be attributed to the following: (1) Local anesthesia for multiple vertebral PKP is often inadequate, causing patients to experience severe pain during the operation, which significantly reduces the patient subjective satisfaction; (2) Patients with m-OVFs often have a rib fracture, humerus fracture, or intertrochanteric fracture simultaneously. Being in a prone position for an extended duration leads to perceptible body position-related discomfort, leading to patient dissatisfaction with preoperative pain management; (3) During PKP under local anesthesia, patients experience high local muscle tension. If the puncture cannot be achieved at one time, the puncture point and puncture angle will be difficult to adjust. Repeatedly adjusting the puncture angle increases the damage to surrounding soft tissue and nerve injury. (4) Often, patients under local anesthesia for PKP do not need time to recover from anesthesia after the operation, and patients can prematurely leave the bed, which shortens the time of wound repair. This study found that the vertebral height and kyphosis in the two groups significantly improved after the operation; however, the improvement rates in the vertebral height and local kyphosis Cobb angle were significantly higher in the general anesthesia group than in the local anesthesia group. The reasons could be as follows: (1) Under general anesthesia, the muscles around the spine become relaxed, and muscle relaxants can significantly reduce muscle tension, which facilitates the recovery of fracture vertebral height and correction of kyphosis; (2) Under general anesthesia, the operation is not affected by frequent changes in body position, unpredictable severe pain, and abrupt fluctuation of vital signs. Focus on precise positioning, targeted puncture, balloon dilatation, and bone cement injection is enhanced, which is conducive to the recovery of the vertebral height and kyphosis correction; (3) Under general anesthesia, patients do not suffer from unbearable pain due to a sudden increase in pressure in the vertebral body during balloon dilatation, which is conducive to injecting larger volumes of bone cement into each vertebral body and promoting the recovery of the vertebral height.

In this study, the incidence of bone cement leakage, nerve complications, vertebral infection, cognitive dysfunction, and cardiopulmonary system complications is similar to those reported in the previous literature [24–26]. The incidence of bone cement leakage and nerve complications was higher in the general anesthesia group than in the local anesthesia group, but no significant difference between the two groups was indicated. In the previous literature, one of the advantages of PKP under local anesthesia is that it can interact with patients during the operation, which can effectively prevent neurological complications. However, the intraoperative interaction can only predict but not prevent the occurrence of spinal cord nerve injury. The surgeon should improve their surgical skills, optimize bone cement injection technology, and improve the accuracy of intraoperative C-arm fluoroscopy as the preferred choice to prevent bone cement leakage and spinal cord nerve injury. In this study, we used PKP under general anesthesia for m-OVFs. During the operation, the mean arterial pressure and heart rate were effectively maintained, and an effective lung ventilation control strategy was adopted. However, 7 patients still suffered from cognitive dysfunction even after the operation, which could be related to primary cardiovascular and cerebrovascular diseases in the elderly, organ dysfunction resulting in the slow metabolism of narcotic drugs, cerebral perfusion pressure, and intraoperative or postoperative hypoxemia. Although no significant difference was indicated between the two groups, cognitive dysfunction was still an important complication in elderly patients receiving surgery under general anesthesia. In addition, the incidence of postoperative cardiopulmonary complications in the general anesthesia group was lower than that in the local anesthesia group, which could be mainly caused by the short duration of general anesthesia and low dosage of narcotic drugs, allowing the patients to return to normal function early after the operation and recover their cardiopulmonary function. In this study, 2 patients suffered from postoperative vertebral infection. The postoperative pathological examination results confirmed that suppurative spondylitis was mistakenly diagnosed as OVF, and PKP was performed. If elderly patients have multiple vertebral body lesions simultaneously, traditional MRI cannot exclude spinal bone tumors and infectious lesions before PKP surgery. In recent years, diffusion tensor imaging and diffusion-weighted imaging, developed based on MRI, present unique advantages in distinguishing purulent spondylitis, spinal tumors, OVF, and so on [27–29]. All patients included in this study were injected with bone cement via unilateral puncture balloon dilatation during the operation. Precise puncture positioning was performed before the operation, and the pressure for balloon expansion pressure was gradually increased. Each patient only needed a set of operation-related equipment, which substantially reduced the medical expenses of the patients. However, the medical costs incurred by the general anesthesia group was still $500 more than that incurred by the local anesthesia group, on average. The difference in medical expenses was also one of the important indexes for the selection of the type of anesthesia.

This study has several limitations. First, inherent limitations for a retrospective study were not avoided; prospective studies should be improved to verify the conclusions in the future. Second, methods for evaluating clinical outcomes, such as cost–utility analysis, were not applied in this study. Lastly, this study failed to exclude the effect of the comorbidities of the included cases on the study results.

## Conclusion

When PKP for the treatment of m-OVFs is performed simultaneously, general anesthesia can more effectively maintain the stability of vital signs such as mean arterial pressure and heart rate, reduce the radiation exposure times of the patients and operators, and shorten the operative time. Regardless of the type of anesthesia, a satisfactory clinical effect can be achieved without an increase in the incidence of complications. The pain improvement rate, vertebral height recovery, and kyphosis correction effect are better in the general anesthesia group than in the local anesthesia group. For elderly patients about to receive PKP under general anesthesia for m-OVFs, effective measures should be observed to prevent cognitive dysfunction. Another factor to consider when choosing the type of anesthesia is medical expenses.

## Data Availability

Data will be available upon request to the first author ZS.

## Abbreviations

PKP: Percutaneous kyphoplasty
m-OVFs: Multiple osteoporotic vertebral fractures
VAS: Visual analog scale
AVH: Anterior vertebral height
MVH: Middle vertebral height
KA: Kyphotic angle
OVF: Osteoporotic vertebral fractures
MRI: Magnetic resonance imaging
ASIA: American Spinal Cord Injury Association
